# Agronomic and Metabolomic Side-Effects of a Divergent Selection for Indol-3-Ylmethylglucosinolate Content in Kale (*Brassica oleracea* var. *acephala*)

**DOI:** 10.3390/metabo11060384

**Published:** 2021-06-14

**Authors:** Jorge Poveda, Pablo Velasco, Antonio de Haro, Tor J. Johansen, Alex C. McAlvay, Christian Möllers, Jørgen A.B. Mølmann, Elena Ordiales, Víctor M. Rodríguez

**Affiliations:** 1 Institute of Agrobiotechnology, Public University of Navarre, 31006 Pamplona, Spain; jorge.poveda@unavarra.es; 2 Mision Biologica de Galicia (MBG-CSIC), 36143 Pontevedra, Spain; pvelasco@mbg.csic.es; 3 Institute of Sustainable Agriculture (CSIC), 14004 Córdoba, Spain; adeharobailon@ias.csic.es; 4 Norwegian Institute of Bioeconomy Research (NIBIO), P.O. Box 115, NO-1431 Ås, Norway; tor.johansen@nibio.no (T.J.J.); jorgen.molmann@nibio.no (J.A.B.M.); 5 Institute of Economic Botany, The New York Botanical Garden, New York, NY 10458, USA; amcalvay@nybg.org; 6 Department of Crop Science, Georg-August-Universität Göttingen, 37075 Göttingen, Germany; cmoelle2@gwdg.de; 7 Centro Tecnológico Nacional Agroalimentario, 06195 Badajoz, Spain; eordiales@ctaex.com

**Keywords:** glucosinolates, kale, abiotic stress, phenolics, flavonoids

## Abstract

*Brassica oleracea* var. *acephala* (kale) is a cruciferous vegetable widely cultivated for its leaves and flower buds in Europe and a food of global interest as a “superfood”. *Brassica* crops accumulate phytochemicals called glucosinolates (GSLs) which play an important role in plant defense against biotic stresses. Studies carried out to date suggest that GSLs may have a role in the adaptation of plants to different environments, but direct evidence is lacking. We grew two kale populations divergently selected for high and low indol-3-ylmethylGSL (IM) content (H-IM and L-IM, respectively) in different environments and analyzed agronomic parameters, GSL profiles and metabolomic profile. We found a significant increase in fresh and dry foliar weight in H-IM kale populations compared to L-IM in addition to a greater accumulation of total GSLs, indole GSLs and, specifically, IM and 1-methoxyindol-3-ylmethylGSL (1MeOIM). Metabolomic analysis revealed a significant different concentration of 44 metabolites in H-IM kale populations compared to L-IM. According to tentative peak identification from MS interpretation, 80% were phenolics, including flavonoids (kaempferol, quercetin and anthocyanin derivates, including acyl flavonoids), chlorogenic acids (esters of hydroxycinnamic acids and quinic acid), hydroxycinnamic acids (ferulic acid and *p*-coumaric acid) and coumarins. H-IM kale populations could be more tolerant to diverse environmental conditions, possibly due to GSLs and the associated metabolites with predicted antioxidant potential.

## 1. Introduction

Crops belonging to the genus *Brassica* are among the top ten most agronomically and economically important vegetable species in the world. These crops show high morphological and agronomic diversity and are cultivated mainly in temperate regions of the Northern Hemisphere [1]. Kale (*Brassica oleracea* var. *acephala*) is a leafy vegetable crop that is becoming popular as a “superfood”, due to its nutritional value (rich in Ca^2+^, folate, riboflavin, vitamins C, K and A), phytochemical composition (including polyphenols, glucosinolates, terpenoids and carotenoids), and its high anticarcinogenic and antioxidant potential [2]. Among *Brassica* phytochemicals, glucosinolates (GSLs)—sulfur compounds derived from amino acids [3]—are the most well-studied compounds. A primary function of GSLs in plants includes defense against pathogens and pests [4,5]. Inside of cells GSLs are chemically stable but, upon cell disruption due to tissue damage, GSLs are exposed to the activity of myrosinase and associated proteins, resulting in glucosinolate hydrolysis products (GHPs) [3]. There are numerous examples of the GHPs antimicrobial activity against plant pathogens, through different mechanisms such as affecting metabolic pathways or cellular structures [6,7]. Against herbivores, mainly insects, the mechanism involved in the defensive capacity of GHPs is direct toxicity by ingestion [8]. However, it has been described how GSLs can also play a role as attractants of beneficial insects, such as pollinators [9] or parasitoids [10], as well as insect-pest of *Brassica* crops [11].

Although GSLs are well-studied in their fundamental role in biotic interactions, there is a lack of understanding of the role of these compounds in other physiological processes. Some evidence indicates that GSLs play a key role as signaling compounds for flowering time, stomatal closure or water transport and may affect auxin signaling [12,13,14]. These alternative roles suggest that GSLs may play a role in adaptation of GSL-containing plants to different environments. Consistent with this possibility, several studies report that abiotic stress conditions such as drought, salinity and extreme temperatures, can impact GSL accumulation in *Brassica* plants (for review see [15]). Bonasia et al. [16] also show that in wild rocket (*Diplotaxis tenuifolia*), GSL content is affected by growing seasons, being higher when plants are cultivated during a winter–spring season. Interestingly, plants with higher GSL content also show a higher yield. In kale, it was reported that exposure to different temperatures significantly modifies the GSL profiles of plant tissues [17]. These findings open a question of whether GSLs may play a role on plant adaptation to different environments.

Different strategies can be used to elucidate the biological effects of GSLs in plants. The most widespread include the use of *Arabidopsis thaliana* mutants [4,18], comparison between species or ecotypes with different GSL profiles [19,20], or the use of populations obtained by divergent selection. Our research group previously carried out three divergent selection programs targeting the three primary GSL compounds in kale. We bred a local Spanish landrace population, MBG-BRS0062, for three cycles and obtained six divergent populations: high and low 3-(methylsulfinyl)propylGSL (3mSOp) content, high and low 2-propenylGSL content and high and low indolylmethylGSL (IM) content [21]. Populations high in IM (H-IM) and low in IM (L-IM) were used in the present study. The differential IM accumulation of these individuals is related to variations in the expression of the *CYP81F2* gene in kale tissues [21]. These populations have previously been used to suggest the role of GSL in the reduction of larval weight in different lepidopteran and aphid pests (*Mamestra brassicae*, *Pieris rapae* and *Brevicoryne brassicae*) [22,23].

In a previous study, no significant differences were reported in the agronomic parameters and profile of GSLs between the kale populations H-IM and L-IM [21]. We decided to cultivate both populations in varied environments to determine how divergent selection for IM content in kale affects agronomic traits evaluated in different environments. Since, it is plausible that a higher accumulation of IM after three cycles of divergent selection may also produce a reorganization of the plant metabolome, we also performed an un-targeted metabolomics analysis to study the role of these changes on plant adaptability.

## 2. Results

### 2.1. Differences in Agronomic Parameters

**Figure 1:**
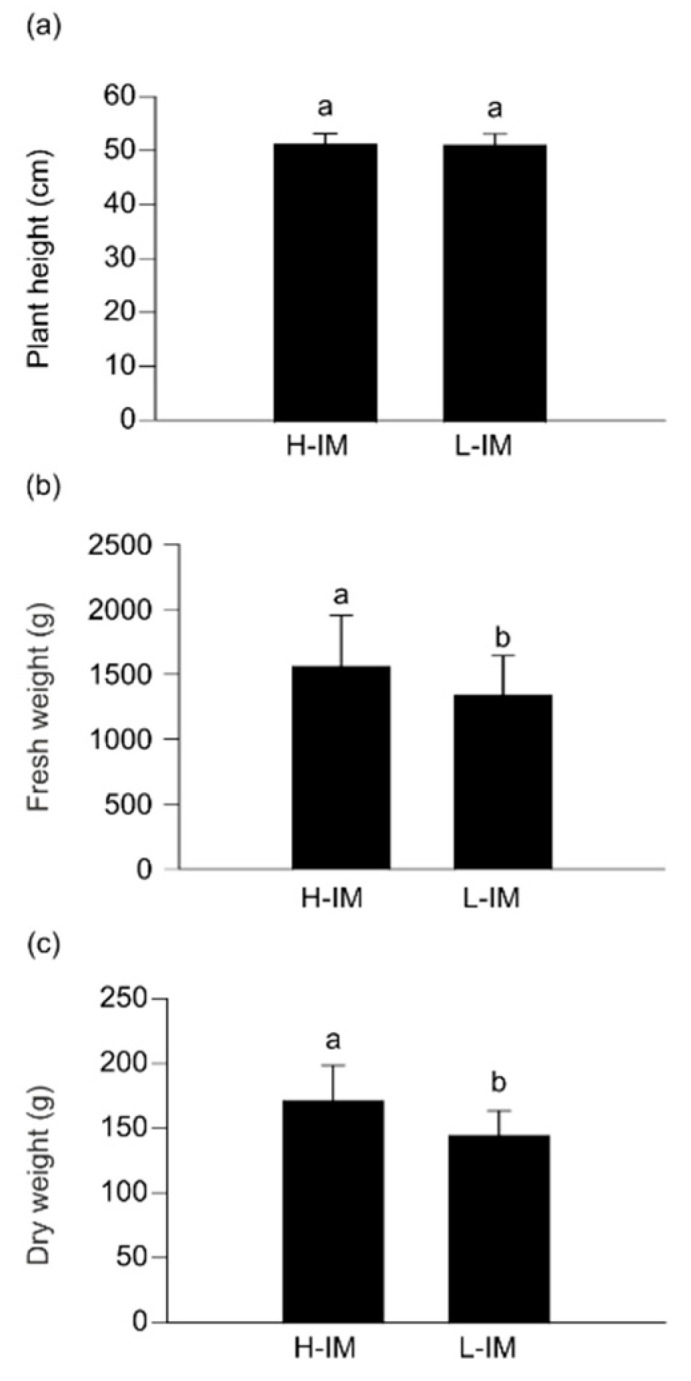
Agronomic parameters in two populations of kale from divergently selected populations with varied indol-3-ylmethylGSL (IM) content (H-IM: High IM content; L-IM: Low IM content). (**a**) Means of plant height of kale populations in all locations. (**b**) Means of fresh weight of 25 leaves of kale populations in all locations. (**c**) Means of dry weight of 25 leaves of kale populations in all locations. Error bars represent ± standard deviation (SD). Within each panel, different letters indicate significant differences between populations (ANOVA, *p*-value ≤ 0.05). Complete ANOVA table results is presented as Supplementary Material (Table S1).

The analysis of variance for plant height showed differences only between locations (L), but not between populations (G) or the L × G interaction. However, there were significant differences between fresh and dry weight between H-IM and L-IM (*p* = 0.0122 and 0.0213, respectively). The mean fresh and dry weights were significantly higher in H-IM (1563 g and 172 g, respectively) than in L-IM (1342 g and 145 g, respectively). The interaction between populations and locations was significant only for fresh weight, but fresh weight was higher in H-IM than L-IM in any location (Figure 1).

### 2.2. GSL Profiles

**Figure 2:**
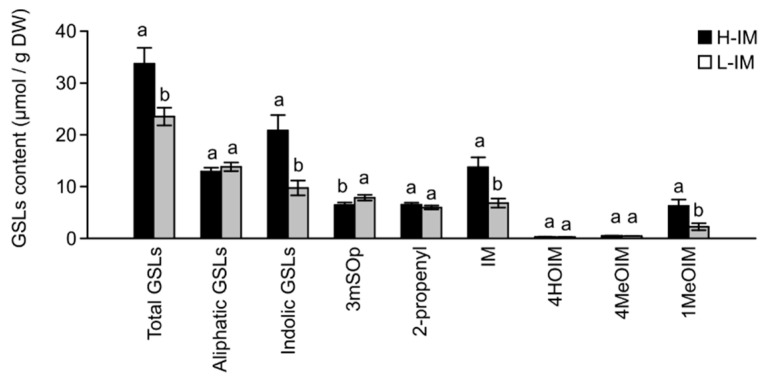
Glucosinolates content in two populations of kale from divergently selected populations with varied indol-3-ylmethylGSL (IM) content (H-IM: High IM content; L-IM: Low IM content). Error bars represent ± standard error (SE). Within each glucosinolate category different letters indicate significant differences between divergent populations (ANOVA, *p*-value ≤ 0.05). Complete ANOVA table results is presented as Supplementary Material (Table S2). Abbreviations: GSLs: Glucosinolates, 3mSOp: 3-(methylsulfinyl)propylGSLs, 2-propenyl: 2-propenylGSL, IM: indol-3-ylmethylGSL, 4HOIM: 4-hydroxyindol-3-ylmethylGSL, 4MeOIM: 4-methoxyindol-3-ylmethylGSL and 1MeOIM: 1-methoxyindol-3-ylmethylGSL.

Our results indicated that the IM content was significantly higher in the H-IM population than in the L-IM population (Figure 2). The analysis of total GSL content of the divergent populations indicated significantly larger amounts in the H-IM (34.1 µmol/g dry weight) population than in the L-IM population (24.3 µmol/g dry weight) (*p* < 0.01). This higher levels of GSLs are mainly due to the accumulation of indole GSLs (*p* < 0.01) in the H-IM population (21.4 µmol/g dry weight) since we did not observe differences between both populations for total aliphatic GSLs content (*p* = 0.0989). Aside from IM, we only observed a significant increase on the accumulation of 1MeOIM (*p* < 0.01) in the H-IM population (6.6 µmol/g dry weight) and a decrease on the accumulation of 3mSOp (6.4 µmol/g dry weight) (*p* = 0.0008) (Figure 2), indicating that the selection method was quite specific in increasing IM content.

### 2.3. Metabolomic Profiles

**Figure 3:**
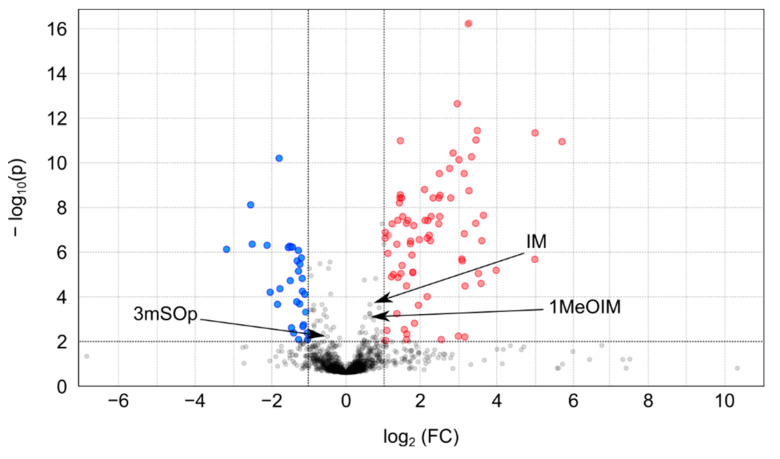
Volcano plot representing the detected features in the non-targeted metabolomic analysis. The y-axis represents the negative decade logarithm of the significance value (*FDR*) and the x-axis represents the log_2_ of fold change (H-IM vs. L-IM). Levels of features with a −log_10_(*p*) ≤ 1.3 and a $\left|log_{2}\left(FC\right)\right|$ ≥ 1 are considered to be differentially accumulated in both populations. Significantly up-regulated features are represented by red circles and down-regulated features are represented by blue circles. Grey circles represent insignificant features. Positions of significant GSLs in the target analysis are represented by arrows (3mSOp: 3-(methylsulfinyl)propylGSL (log_2_(FC) = −0.39; −log_10_(p) = 1.91); IM: indol-3-ylmethylGSL (log_2_(FC) = 0.36; −log_10_(p) = 2.72); 1MeOIM: 1-methoxyindol-3-ylmethylGSL (log_2_(FC) = 0.54; −log_10_(p) = 1.55)).

In order to identify the metabolomic changes that could take place in the kale population due to divergent selection on IM content, we performed a non-targeted metabolomics analysis. Statistical univariate analyses reported 109 features that were differentially accumulated in the H-IM and L-IM populations (Figure 3). Data was then hand-filtered, taking into account retention time and correlation coefficients to remove features that were most likely due to in-source fragmentation of metabolites. Ultimately, 67 features of these were considered to be true metabolites (30 detected in negative and 37 in positive ionization modes) (Table 1). Forty-four of these metabolites had increased concentration in H-IM populations compared to L-IM populations, while 23 metabolites had decreased concentrations. IM was not among the selected metabolites since though showed significantly higher levels in the H-IM population vs. L-IM (*p* value = 0.001) in the metabolomics analysis, did not fit the fold change condition.

**Figure 4:**
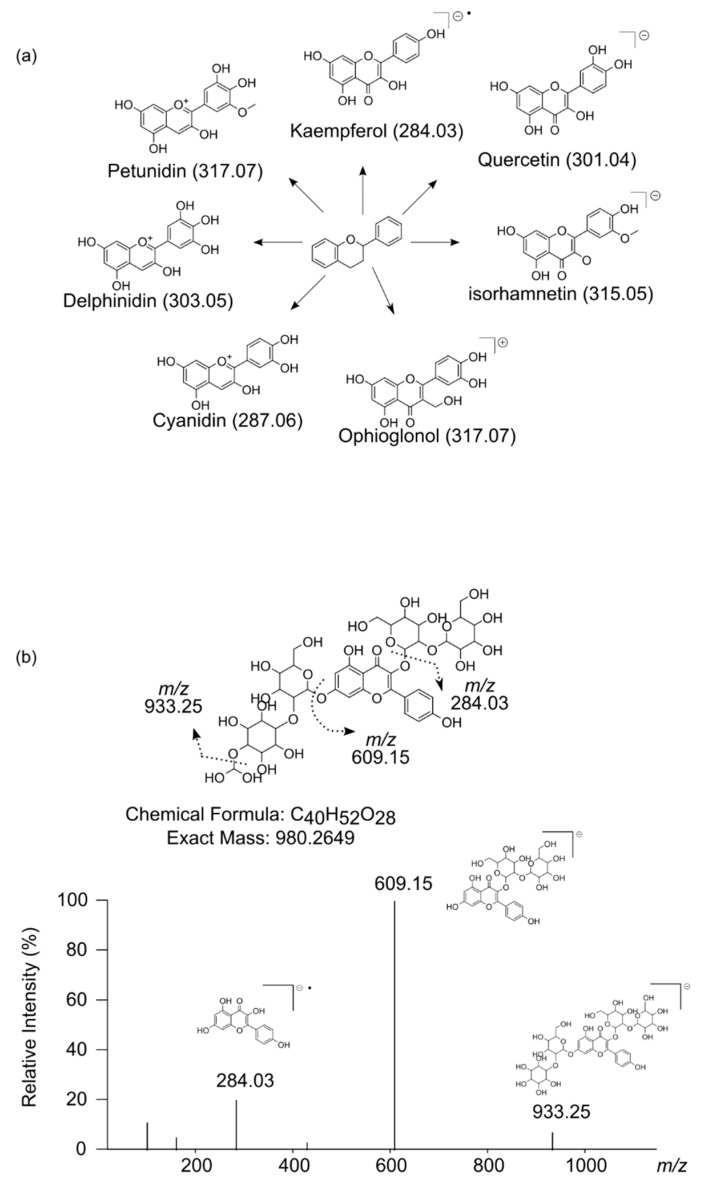
Identification of flavonoids based on MS/MS fragmentation patterns. (**a**) Ions corresponding to aglycones of the different flavonoids identified in this work. The central structure corresponds to the basic skeleton of flavonoids. The mass of each flavonoid aglycone is reported in brackets. (**b**) Proposed structure for the *m*/*z* 980.2649. Dot lines indicate the hypothetical pattern of fragmentation of the proposed molecule based on the experimental MS/MS mass spectrum obtained.

When possible, a molecular formula was assigned to each metabolite based on the exact mass and the isotopic pattern. Tentative identification was performed based on the molecular formula and MS/MS fragmentation pattern. We were able to tentatively assign compound names to 52 out of 67 metabolites. The majority of these compounds are classified as phenolics (80%). Among them, 70% are classified as flavonoids (kaempferol, quercetin and anthocyanins derivates, including acyl flavonoids), 18% as chlorogenic acids (esters of hydroxycinnamic acids and quinic acid), 6% as hydroxycinnamic acids (ferulic acid and *p*-coumaric acid) and 6% as coumarins. Flavonoids were identified on the bases of the aglycone fragment (Figure 4a). Deviation of the aglycone *m*/*z* can be observed in some signals on Table 1 (i.e., 285.04 or 284.03 on kaempferol glycosides) due to homolytic or heterolytic fragmentation. The homolytic fragmentation of flavonoid glycosides produces a radical aglycone ion [Y_0_−H]^−•^ (*m*/*z* 284.03 for kaempferol), whereas the heterolytic fragmentation produces an aglycone fragment ion [Y_0_]^−^ (*m*/*z* 285.04 for kaempferol).

We propose the tentatively identification of the ion at *m*/*z* 980.26 (RT: 11.2 min) as kaempferol-sophoroside-(dihydroxymethoxy)-sophoroside (Table 1). The most abundant fragment of this compound corresponds to a kaempferol-sophoroside (*m*/*z* 609.15) (Figure 4b). The kaempferol aglycone was also confirmed by the presence of a peak at *m*/*z* 284.03. Identification of a kaempferol-3-*O*-sophoroside-7-*O*-sophoroside (*m/z* 934.25) was previously reported in *B. oleracea* [38]. The neutral loss of *m*/*z* 371.11 (980.26 → 609.15) may indicate the loss of an anhydrohexose attached to a dihydroxymethoxy cyclohexane. Finally, the neutral loss of *m*/*z* 47.01 (980.26 → 933.25), supports the hypothesis of the presence of a dihydroxymethoxy group. This is, however, just a proposed structure and a conclusive elucidation will require further analysis.

Significant metabolites are evenly distributed between the groups of compounds with higher and lower concentration in the H-IM population compared to the L-IM population. The only exception is the group of coumarins that showed a higher concentration in the H-IM population. Aside from phenolics, we identified two compounds: kynurenic acid, a product of the kynurenine branch of tryptophan metabolism and an indolylacetyl dihexoside, a carbohydrate derivative.

## 3. Discussion

Changes on the metabolome allow plants to adapt to fluctuations in the environmental conditions. The accumulation of specific metabolites, especially those with antioxidant properties, act as a metabolic buffer under stressful conditions. It has been demonstrated that different environmental and cultivation conditions modify the profile and content of GSLs in *Brassica* crops [39,40]. In general terms, abiotic stresses tend to increase the content of GSLs in these plants, suggesting that GSLs may play a role on plant adaptation to different environments. However, the direct role of these compounds in plant adaptation has yet to be addressed. In this work we used two divergently selection populations (H-IM and L-IM) to study that possible role.

Our results may indicate that the leaf productivity of *Brassica* crops could be directly or indirectly affected by IM content and that this effect is stable across different environments. We observed a higher yield in the H-IM population in the various experimental locations from southern Spain to northern Norway. To the best of our knowledge this is the first time that the possible role of the IM has been studied directly in relation to local adaptation. Interestingly, both populations barely differ in the amount of other GSLs, so differences in yield could be attributed to a great degree to the accumulation of IM. However, it is plausible that a higher accumulation of IM after three cycles of divergent selection may also produce a reorganization of the plant metabolome, that could contribute to increased plant adaptability.

To study the extent of potential metabolome reorganization we performed an untargeted metabolomics analysis. Extraction conditions (80% MeOH), chromatographic setup (reverse-phase UPLC) and ionization interface (ESI) used in our analysis allowed for detection of a wide range of polar and mid-polar metabolites, but with a lack of information about highly polar (elute with the dead volume of the chromatography system) or apolar compounds. With this limitation in mind, our analysis indicate that divergent selection mainly affected phenolic biosynthetic pathways. More than 80% of the metabolites identified were phenols. Previous studies have reported a simultaneous increase of IM and total phenols in various crucifers (*Isatis canescens*, *B. oleracea* var. *italica*, or *B. rapa* ssp. *rapa*) [41,42,43]. However, our study represents the first example of a possible relationship between higher IM content and higher phenolic compound content in kale.

Phenolic compounds constitute a complex group of secondary metabolites that are widespread in the plant kingdom. They have allelopathic, antimicrobial and antioxidant activity in plants [44,45,46] and can be precursors of other secondary metabolites (e.g., hydroxycinnamic acids are precursor of lignin) [47]. It is not surprising that most of the phenolics we identified were flavonoids given that they are the most prominent phenolics in *Brassica* species [24]. Severe stress conditions activate the biosynthesis of flavonoids, which in turn act as an antioxidant system preventing cellular damage. It may be hypothesized that the high levels of IM, a stress-promoted molecule, are perceived by the plant as an indicator of stressful conditions, resulting in the activation of flavonoid biosynthesis. Based on our agronomic results, this is unlikely since plants with an imbalanced defensive response show lower growth rates [48,49]. Some authors suggest a direct or indirect role of flavonoids as growth regulators. Grandmaison et al. [50] reported that flavonoids can regulate cell development by interaction with nuclear proteins. Supporting this idea, Saslowsky et al. [51] demonstrate that the end products of flavonoid biosynthesis are located in the cytoplasm and the nuclei of the tip cells of *Arabidopsis* roots where they can interact with auxin biosynthesis. In vitro analysis shows that in both subcellular compartments, cytoplasmic and nuclear, flavonoids can also interact with actin, regulating its polymerization [52]. This interaction is structure dependent, with flavonols acting as inhibitors and flavanes as stimulators of actin polymerization [52].

We tentatively identified several derivatives of flavonol (quercetin, kaempferol, (iso)rhamnetin) and anthocyanin (cyanidin, petunidin and delphinidin) that accumulated differentially in the H-IM and L-IM populations. Only cyanidin glycosides, along with the group of coumarins, accumulate in a higher extent in the H-IM than in the L-IM population. Curiously, these two groups of compounds have been reported to inhibit plant development [53,54], so further studies will be necessary to elucidate the role of these compounds in kale growth.

## 4. Materials and Methods

### 4.1. Plant Populations

Two divergently selected kale populations, one with high (H-IM) and one with low (L-IM) IM content, were used in this study. These two populations were selected from a local Spanish population (MBG-BRS0062), kept at the *Brassica* germplasm bank at Misión Biológica de Galicia (MBG-CSIC) (Pontevedra, Spain). These populations had been subjected to three selection cycles (details explained in Sotelo et al.) [21].

### 4.2. Growing Conditions and Locations

Kale seeds (H-IM and L-IM) were sown in multipot-trays in a greenhouse. At the 5–6 leaf stage, plants (50 plants/plot) were transplanted into fields in a randomized block design with two replications. Evaluations were performed during the growing season of 2017 in five locations: Pontevedra (PO) (Spain; 42°26′ N, 8°38′ W), Badajoz (BA) (Spain; 38°53′ N, 6°51′ W), Córdoba (CO) (Spain; 37°53′ N, 4°42′ W), Göttingen (GO) (Germany; 51°32′ N, 9°54′’ E) and Tromsø (TR) (Norway; 69°40′ N, 18°56′ E). Transplantation and harvest were carried out on 2 April and 26 September (PO), 24 March and 26 September (BA), 5 April and 29 September (CO), 14 June and 31 August (TR) and 16 May and 5 September (GO), respectively. Cultivation operations, fertilization and weed control were carried out according to local practices and crop requirements.

### 4.3. Agronomic Parameters

Fresh weight was quantified using twenty-five fully developed leaves (7th-8th leaf from the apex) from each plot, harvested randomly. The same leaves were subsequently dried at 70 °C until a constant weight was reached to record the dry mass. Plant height was measured from the soil surface to the base of the upper leaf in 10 plants from each plot.

### 4.4. Biochemical Analysis

For GSLs and non-targeted metabolomics analyses, the 4th leaf from the apex of 15 plants/plot were collected in liquid nitrogen and stored at −80 °C until freeze-dried in a lyophilizer (GAMMA 2-16 LSC plus; Christ, Osterode am Harz, Germany). Samples were mechanically milled to a fine powder in a grinder (Janke and Kunkel A10 mill; IKA-LabortechnikStaufen, Staufen, Germany) before metabolite extraction.

#### 4.4.1. GSLs Analysis

The analysis of the GSL-profiles in the samples was carried out following the methodology described by [55], with some modifications. Twelve milligrams of freeze-dried kale powder was mixed with 400 μL 70 % (*v*/*v*) methanol preheated to 70 °C, 10 μL of PbAc (0.3 M) and 120 μL of ultra-pure water preheated to 70 °C. Before, 20 μL of glucotropaeolin was added as an internal standard. The tubes were shaken in a Microplate incubator (Model OVAN Orbital Midi; OVAN, Badalona, Spain) at 250 rpm for one hour and centrifuged at 3700 rpm for 12 min. Subsequently, 400 µL of the glucosinolate extracts was pipetted into an ion-exchange column with Sephadex DEAE-A25 (Sigma-Aldrich, St. Louis, MO, USA). Desulphation was carried out by adding purified sulphatase (E.C. 3.1.6.1, type H-1 from Helix pomatia) (Sigma-Aldrich, St. Louis, MO, USA) solution. Finally, the desulphated GSLs were diluted in 200 µL of ultra-pure water and 200 µL of 70% methanol and kept frozen for further analyzes.

The chromatographic analyses were carried out on an Ultra-High-Performance Liquid Chromatograph (UHPLC Nexera LC-30AD; Shimadzu, Kyoto, Japan) equipped with a Nexera SIL-30AC injector (Shimadzu, Kyoto, Japan) and one SPDM20A UV/VIS photodiode array detector (Shimadzu, Kyoto, Japan). The UHPLC column was an X Select ^®^HSS T3 (2.5µm particle size, 2.1 × 100 mm i.d.) from Waters (Waters Corporation, Milford, MA, USA) protected with a VanGuard pre-column. The oven temperature was set at 35 °C. GSLs were quantified at 229 nm and were separated by using the following method in aqueous acetonitrile, with a flow of 0.5 mL min^−1^: 1.5 min at 100% H_2_O, an 11 min gradient from 5% to 25% (*v*/*v*) acetonitrile, 1.5 min at 25% (*v*/*v*) acetonitrile, a minute gradient from 25% to 0% (*v*/*v*) acetonitrile and a final 3 min at 100% H_2_O. Specific GSLs were identified by comparing retention times and UV spectra with standards. GSLs standards were purchase from Phytoplan (Diehm & Neuberger GmbH, Heidelberg, Germany). Calibration equations were made with at least five data points, from 0.08 to 1.3 nmol for 3mSOp (y = 8.16 × 10^−6^ x; R^2^ = 0.99), from 0.10 to 1.56 nmol for 2-propenyl (y = 1.06 × 10^−5^ x; R^2^ = 1.00), from 0.07 to 1.19 nmol for IM (y = 3.42 × 10^−6^ x; R^2^ = 1.00), from 0.07 to 1.09 nmol for 4MeOIM (y = 2.98 ×1 0^−6^ x; R^2^ = 0.99) and from 0.07 to 1.09 nmol for 1MeOIM (y = 1.5 × 10^−6^ x; R^2^ = 1.00). The standard curve of IM was used to quantify the amount of 4HOIM using a response factor of 1.

#### 4.4.2. Metabolomic Analysis

Freeze-dried powder (50 mg) was dissolved in 500 mL of 80% aqueous methanol and then sonicated for 15 min. After centrifugation for 10 min (16,000× *g*, at room temperature), the extract was filtered through a 0.20 µm micropore PTFE membrane and placed in vials for further analysis. For metabolomic composition analysis we used ultra–performance liquid chromatography coupled with electrospray ionization quadrupole (Thermo Dionex Ultimate 3000 LC; Thermo Fisher Scientific, Waltham, MA, USA) time–of–flight mass spectrometry (UPLC–Q–TOF–MS/MS) (Bruker Compact™) with a heated electrospray ionization (ESI) source. Chromatographic separation was performed in an Intensity Solo 2 C18 column (2.1× 100 mm 1.7 µm pore size; Bruker Daltonics, Billerica, MA, USA) using a binary gradient solvent mode consisting of 0.1% formic acid in water (solvent A) and acetonitrile (solvent B). The following gradient was used: 3 % B (0-4 min), from 3% to 25 % B (4–16 min), from 25 to 80% B (16–25min), from 80 to 100% B (25–30 min), hold 100% B until 32 min, from 100% to 3% B (32–33 min), hold 3% B until 36 min. The injection volume was 5 µL, the flow rate was established at 0.4 mL/min and column temperature was controlled at 35 °C. MS analysis was operated in spectra acquisition range from 50 to 1200 *m/z*. Both polarities (±) of ESI mode were used under the following specific conditions: gas flow 9 L/min, nebulizer pressure 38 psi, dry gas 9 L/min, and dry temperature 220 °C. Capillary and end plate offset were set to 4500 and 500 V, respectively. The instrument was calibrated externally with a calibration solution of 1mM sodium formate/acetate in iPrOH/H_2_O 50/50 with 0.2% formic acid directly infused to the source. Before sample injections, LC-qTOF system stability was tested by three consecutive injections of chloramphenicol (ESI–mode; ΔRT= 0.02 min; Δ*m*/*z* = 0.002) and triphenyl phosphate (ESI + mode; ΔRT= 0.02 min; Δ*m*/*z* = 0.001). The calibration solution was injected at the beginning of each run and all the spectra were calibrated prior to statistical analysis. MS/MS analysis was performed based on the previously determined accurate mass and RT and fragmented by using different collision energy ramps to cover a range from 15 to 50 eV. The algorithm T–Rex 3D from the MetaboScape 4.0 software (Bruker Daltonics, Billerica, MA, USA) was used for peak alignment and detection.

#### 4.4.3. Statistical Analysis

Parametric statistical analysis was performed using the GLM procedure of SAS 9.4 (SAS Institute Inc., Cary, NC, USA) for agronomic traits and GSLs content. Populations were considered fixed effects and locations were considered random effects. A post hoc ANOVA analysis was performed using the Fisher’s protected least significant difference (LSD) at *p* ≤ 0.05.

Statistical analysis of metabolomic data was performed using the web-based software Metaboanalyst [56]. In order to remove non-informative variables, data were filtered using the interquantile range filter (IQR). Moreover, Pareto variance scaling was used to remove the offsets and adjust the importance of high- and low-abundance ions to an equal level. The resulting three-dimensional matrix (peak indices, samples and variables) was further subjected to statistical analysis. Univariate analysis (one-way ANOVA) with a *p* value ≤ 0.05 was carried out to find differentially expressed metabolites. Using the Volcano Plot (VP) approach, which measure differentially accumulated metabolites based on *t*-statistics and fold changes simultaneously, we also highlighted the metabolites with a $\left|\log_{2}\left(\mathrm{FC}\right)\right|$ ≥ 1 and statistically significant difference (FDR ≤ 0.05) between populations.

#### 4.4.4. Tentative Metabolite Identification

Tentative compound identification was performed using accurate mass metabolites reported in different publicly available databases such as METLIN, KEGG, Pubchem, HMDB and Plant Metabolic Network. Additionally, further partial identification of the most significant metabolites was made by comparison of MS/MS fragmentation patterns against reference compounds found in previously mentioned databases and bibliography on plants of the *Brassicaceae* family.

## 5. Conclusions

We reported a higher yield of H-IM populations of kale across different environments compared to L-IM populations indicating a potentially greater adaptive capacity of the H-IM populations to varied contexts, as measured by a higher production of foliar biomass. The GSL profiles analysis showed a higher content in indole GSLs in H-IM populations, previously described secondary metabolites which are thought to impart higher tolerance to abiotic stresses such as salinity [57] or drought [58]. The H-IM populations of kale had higher concentrations of compounds, which tentatively can be predicted to have antioxidant potential that may contribute to tolerance of abiotic stresses by reducing the generation of reactive oxygen species [59]. The high indole GSL content and the accumulation of other secondary metabolites may give the H-IM populations of kale an improved adaptive capacity under varied environmental conditions, which may be responsible for an observed higher yield of the high indole GSL population.

## Figures and Tables

**Table 1:** Tentative identification of metabolites with a $\left|log_{2}\left(FC\right)\right|$ ≥ 1 and statistically significant difference (FDR ≤ 0.05) between populations (High-Indol-3-ylmethylGSL vs. Low-Indol-3-ylmethylGSL content). Metabolites are sorted by ionization mode. References to the identification of compounds in the *Brassicaceae* plant family is included when available. Compounds in bold: fragmentation spectra match with authentic standard. Underlined compounds: retention time match with authentic standard.

*m/z*	Neutral Mass	Ionization	RT (Min)	Log_2_(FC)	Molecular Formula	Theoretical Mass	Mass Deviation (ppm)	Fragments	Tentative Identification
**A. Peaks detected in negative ionization mode**									
337.0937	338.1010	[M−H]^−^	10.1	2.1	C_16_H_18_O_8_	338.1002	2.514	119.05, 163.04, 191.06	coumaroylquinic acid isomer 1 [24]
771.1991	772.2064	[M−H]^−^	10.3	−1.1	C_33_H_40_O_21_	772.2062	0.176	283.04, 609.17, 255.03, 422.07, 446.10	kaempferol sophoroside-hexoside [25]
675.1938	338.1005	[2M−H]^−^	10.4	2.2	C_16_H_18_O_8_	338.1002	1.035	163.04, 337.09, 191.06	coumaroylquinic acid isomer 2 [24]
325.0937	326.1010	[M−H]^−^	10.8	1.8	C_15_H_18_O_8_	326.1007	0.889	119.05, 163.04	coumaroylglucoside [26]
489.1252	980.2650	[M−2H]^−2^	11.2	−1.2	C_40_H_52_O_28_	980.2645	0.490	609.15, 284.03, 933.25, 101.02	kaempferol sophoroside-(dihydroxymethoxy) sophoroside ^1^
628.1641	629.1714	[M−H]^−^	11.9	−1.0				466.11, 284.03, 161.02	kaempferol hexoside derivative or isomer
625.1416	626.1488	[M−H]^−^	11.9	3.1	C_27_H_30_O_17_	626.1483	0.854	299.03, 271.04	quercetin sophoroside [27]
337.0932	338.1005	[M−H]^−^	12.0	1.5	C_16_H_18_O_8_	338.1002	0.917	173.05, 119.05, 93.03, 163.04	coumaroylquinic acid isomer 3 [24]
569.1518	1140.3181	[M−2H]^−2^	12.6	-1.8	C_50_H_60_O_30_	1140.3170	0.991	488.12, 407.09, 815.20, 205.05, 284.03	kaempferol sinapoylsophoroside-gentobioside [28]
635.1728	1272.3601	[M−2H]^−2^	12.7	−1.3	C_55_H_68_O_34_	1272.3592	0.684	473.12, 635.17, 947.24, 119.04, 161.05, 263.07, 323.10	kaempferol feruloylpentaglucoside [29]
609.1463	610.1536	[M−H]^−^	12.8	2.1	C_27_H_30_O_16_	610.1534	0.295	477.09, 285.04	kaempferol dihexoside [30]
554.1465	1110.3075	[M−2H]^−2^	12.8	−1.3	C_49_H_58_O_29_	1110.3064	1.013	473.12, 392.09, 785.19, 284.03, 175.04, 609.14	kaempferol feruloylsophoroside-cellobioside [28]
337.0935	338.1008	[M−H]^−^	12.9	1.1	C_16_H_18_O_8_	338.1002	1.863	173.04, 93.03, 119.05, 163.04, 111.04	coumaroylquinic acid isomer 4 [24]
755.2047	756.2119	[M−H]^−^	13.2	1.8	C_33_H_40_O_20_	756.2113	0.847	283.04, 255.04, 609.17, 227.05, 430.10	kaempferol gentiobioside-rhamnoside isomer 1 [31]
337.0935	338.1014	[M−H]^−^	13.9	1.5	C_16_H_18_O_8_	338.1002	3.668	191.05, 119.05, 163.04, 127.04	coumaroylquinic acid isomer 5 [24]
771.2003	772.2076	[M−H]^−^	14.2	3.6	C_33_H_40_O_21_	772.2062	1.782	447.09, 625.14, 301.03	quercetin-(rhamnosylhexoside)-hexoside or isomer
625.142	626.1493	[M−H]^−^	14.3	−1.2	C_27_H_30_O_17_	626.1483	1.526	300.03, 179.0, 445.08, 463.09	quercetin dihexoside [32]
609.1468	610.1540	[M−H]^−^	14.3	1.6	C_27_H_30_O_16_	610.1534	1.048	446.08, 283.02, 463.09, 301.03	quercetin hexoside-rhamnoside [33]
755.2044	756.2117	[M−H]^−^	15.0	2.9	C_33_H_40_O_20_	756.2113	0.517	609.15, 431.10, 285.04	kaempferol gentiobioside-rhamnoside isomer 2 [34]
593.1518	594.1584	[M−H]^−^	15.2	5.0	C_27_H_30_O_15_	594.1585	0.143	430.09, 447.09, 285.04	**kaempferol rutinoside or isomer**
639.1569	640.1642	[M−H]^−^	15.4	−3.2	C_28_H_32_O_17_	640.1640	0.305	314.04, 459.09, 609.15	(iso)rhamnetin-dihexoside
623.1625	624.1705	[M−H]^−^	15.5	3.6	C_28_H_32_O_16_	624.1690	2.339	461.11, 477.10, 315.05	(iso)rhamnetin-rhamnosylhexoside
657.1752	1316.3649	[M−2H]^−2^	17.0	−1.3	C_60_H_68_O_33_	1316.3643	0.437	576.14, 284.03, 175.04, 205.05	kaempferol-(feruloyl)(sinapoyl)-trihexoside-hexoside or isomer 1
709.1998	710.2071	[M−H]^−^	17.1	1.6	C_32_H_38_O_18_	710.2058	1.816	485.13, 161.03, 223.06, 179.04	kaempferol derivative or isomer
657.1745	1316.3641	[M−2H]^−2^	17.2	−1.5	C_60_H_68_O_33_	1316.3643	0.141	576.15, 284.03, 205.05, 175.04, 947.26	kaempferol-(feruloyl)(sinapoyl)-trihexoside-hexoside or isomer 2
415.1977	416.2050	[M−H]^−^	17.6	1.8	C_20_H_32_O_9_	416.2046	0.781	44.99, 71.02, 113.03, 101.02	nicotinic acid hexoside derivative
709.4687	710.4760	[M−H]^−^	28.7	−1.8					
683.4655	684.4728	[M−H]^−^	29.5	−1.8					
683.4659	684.4732	[M−H]^−^	29.8	−2.1					
709.4807	710.4880	[M−H]^−^	29.8	−2.0					
**B. Peaks detected in positive ionization mode**									
110.0702	109.0629	[M+H]^+^	0.8	1.1				47.77	
190.0502	189.0431	[M+H]^+^	9.7	−1.5	C_10_H_7_NO_3_	189.0426	2.412	116.05, 162.05, 89.04, 144.04	**kynurenic acid**
361.0893	360.0820	[M+H]^+^	10.1	1.1				147.05, 167.06, 91.05	feruloyl derivative
147.0433	146.0360	[M+H]^+^	10.1	1.4	C_9_H_6_O_2_	146.0368	5.478	65.04, 91.05	**coumarin isomer 1** ^2^
449.1079	448.1006	[M+H]^+^	10.2	−1.2	C_21_H_20_O_11_	448.1006	0.036	305.07, 287.05	cyanidin-hexoside or isomer
339.1074	338.1001	[M+H]^+^	10.4	1.2	C_16_H_18_O_8_	338.1002	0.177	147.04, 119.05, 91.05	coumaroylquinic acid isomer 2 [24]
361.0894	360.0821	[M+H]^+^	10.4	1.1				147.04, 167.05, 140.99, 91.05	feruloyl derivative
803.2232	802.2160	[M+H]^+^	11.4	−2.5	C_34_H_42_O_22_	802.2168	1.022	317.06, 479.12	ophioglonol-dihexoside-hexoside or isomer 1
147.0429	146.0356	[M+H]^+^	12.1	1.9	C_9_H_6_O_2_	146.0368	8.217	65.04, 91.05, 63.03, 55.05	**coumarin isomer 2** ^2^
773.2121	772.2049	[M+H]^+^	12.6	1.5	C_33_H_41_O_21_^+^	772.2062	1.740	287.05, 303.05, 449.10	cyanidin sophoroside-hexoside [35]
147.0440	146.0367	[M+H]^+^	12.9	1.7	C_9_H_6_O_2_	146.0368	0.342	91.05, 65.04, 53.04	**coumarin isomer 3** ^2^
803.2232	802.2160	[M+H]^+^	13.1	−2.5	C_34_H_42_O_22_	802.2168	1.022	317.06, 85.03, 145.05, 479.12	ophioglonol-dihexoside-hexoside or isomer 2
757.2173	756.2100	[M+H^]+^	13.2	2.5	C_33_H_40_O_20_	756.2113	1.744	287.05, 85.03, 433.11, 145.05	cyanidin-rhamnosylhexoside-hexoside or isomer 1
949.2599	948.2527	[M+H]^+^	13.4	2.8	C_43_H_48_O_24_	948.2536	0.938	177.05, 287.05, 339.10, 449.10	cyanidin-(feruloyldihexoside)-hexoside or isomer
233.1654	232.1581	[M+H]^+^	13.5	2.5					
979.2703	978.2634	[M+H]^+^	13.5	3.1	C_44_H_51_O_25_^+^	978.2641	0.721	287.05, 369.11, 449.11, 611.16	cyanidin-(sinapoyldihexoside)-hexoside or isomer
919.2493	918.2420	[M+H]^+^	13.7	3.1	C_42_H_46_O_23_	918.2430	1.040	163.04, 287.05, 325.09, 307.08	cyanidin-(coumaroyldihexoside)-hexoside [36]
147.0393	146.0320	[M+H]^+^	13.9	1.3				91.05, 65.04,55.06, 53.04	
773.2127	772.2054	[M+H]^+^	14.1	3.6	C_33_H_40_O_21_	772.2062	1.080	303.05, 85.03, 145.05, 287.05, 449.11	delphinidin-rutinoside-hexoside [36]
963.2752	962.2679	[M+H]^+^	14.2	2.5	C_44_H_51_O_24_^+^	962.2692	1.319	287.05, 369.12, 433.11, 207.07	cyanidin-(sinapoylhexoside)-rhamnosylhexoside or isomer
465.1027	464.0954	[M+H]^+^	14.3	−1.1	C_21_H_20_O_12_	464.0955	0.086	303.05, 85.03, 127.04	**delphinidin-hexoside isomer** [37]
611.1600	610.1527	[M+H]^+^	14.3	2.1	C_27_H_30_O_16_	610.1534	1.082	303.05, 287.05, 85.03, 127.04, 449,11	cyanidin-dihexoside or isomer 1
633.2012	632.1940	[M+H]^+^	14.4	2.2	C_27_H_36_O_17_	632.1953	2.048	147.04, 165.05, 127.04, 85.03, 309.10	feruloyl derivative
933.2648	932.2575	[M+H]^+^	14.5	3.0	C_43_H_48_O_23_	932.2586	1.260	177.05, 287.05, 339.11, 321,10, 127.04, 433.11	cyanidin-(feruloylhexoside)-rhamnosylhexoside or isomer
903.2545	902.2472	[M+H]^+^	14.7	3.2	C_42_H_47_O_22_^+^	902.2481	0.964	147.04, 287.05, 309.10, 291.09, 433.11	cyanidin-(coumaroylhexoside)-rhamnosylhexoside or isomer
757.2172	756.2099	[M+H]^+^	15.0	3.2	C_33_H_40_O_20_	756.2113	1.889	287.05, 85.03, 127.04, 433.11	cyanidin-rhamnosylhexoside-hexoside or isomer 2
369.1184	368.1111	[M+H]^+^	15.0	−1.4	C_17_H_20_O_9_	368.1107	0.964	175.04, 207.07, 147.04	feruloyl quinic acid isomer 1 [24]
611.1599	610.1526	[M+H]^+^	15.2	−1.5	C_27_H_30_O_16_	610.1534	1.246	287.05, 85.03, 127.04, 97.03, 145.05	cyanidin-dihexoside or isomer 2
394.1136	393.1063	[M+H]^+^	15.3	−1.1					
625.1752	624.1679	[M+H]^+^	15.5	4.0	C_28_H_32_O_16_	624.1690	1.794	317.07, 85.03, 303.05, 127.04, 97.03	petunidin-rhamnosylhexoside or isomer
500.1758	499.1688	[M+H]^+^	15.8	1.6	C_22_H_29_NO_12_	499.1690	0.459	130.07, 85.03, 160.08, 307.10	indolylacetyl dihexoside
468.1501	467.1428	[M+H]^+^	15.8	1.6					
369.1183	368.1110	[M+H]^+^	16.0	−1.1	C_17_H_20_O_9_	368.1107	0.774	175.04, 207.07, 147.04, 119.05	feruloyl quinic acid isomer 2 [24]
517.1547	516.1474	[M+H]^+^	16.5	1.8	C_22_H_28_O_14_	516.1479	0.903	193.05, 161.02, 85.03, 127.04, 69.03, 97.03	caffeoyl quinic acid hexoside or isomer 1
517.1553	516.1479	[M+H]^+^	16.8	1.5	C_22_H_28_O_14_	516.1479	0.038	193.05, 161.02, 85.03, 127.04, 69.03, 97.03	caffeoyl quinic acid hexoside or isomer 2
393.1893	370.1997	[M+Na]^+^	17.6	2.1	C_19_H_30_O_7_	370.1992	1.364		
457.2064	456.1995	[M+H]^+^	18.8	1.9	C_22_H_32_O_10_	456.1996	0.175	191.14, 147.11, 121.07, 93.07, 69.07, 209.16	

^1^ Proposed chemical name based on exact mass and MS/MS fragmentation spectra (please see text and Figure 4b for more details). ^2^ The retention time of authentic coumaric acid standard was 17.7 min, different from all three detected isomers.

## Data Availability

The datasets generated during the current study are available from the corresponding author on reasonable request.

## Supplementary Materials

The following are available online at https://www.mdpi.com/article/10.3390/metabo11060384/s1, Table S1: ANOVA analysis of agronomic parameters in two populations of kale from divergently selected populations with varied indol-3-ylmethylGSL (IM) content (H-IM: High IM content; L-IM: Low IM content), Table S2: ANOVA analysis of glucosinolates GSL content in two populations of kale from divergently selected populations with varied in-dol-3-ylmethylGSL (IM) content (H-IM: High IM content; L-IM: Low IM content). 3mSOp (3-(methylsulfinyl)propylGSL), 2-propenylGSL, IM (indol-3-ylmethylGSL), 4HOIM (hydroxyin-dol-3-ylmethylGSL), 4MeOIM (4-methoxyindol-3-ylmethylGSL), 1MeOIM (1-methoxyindol-3-ylmethylGSL).
